# Fabrication and Characterization of SnO_2_/Graphene Composites as High Capacity Anodes for Li-Ion Batteries

**DOI:** 10.3390/nano3040606

**Published:** 2013-11-15

**Authors:** Abirami Dhanabalan, Xifei Li, Richa Agrawal, Chunhui Chen, Chunlei Wang

**Affiliations:** Department of Mechanical and Materials Engineering, Florida International University, Miami 33174, FL, USA; E-Mails: adhan001@fiu.edu (A.D.); xfli2011@hotmail.com (X.L.); ragra005@fiu.edu (R.A.); cchen012@fiu.edu (C.C.)

**Keywords:** tin oxide, graphene, composites, lithium ion battery, anodes

## Abstract

Tin-oxide and graphene (TG) composites were fabricated using the Electrostatic Spray Deposition (ESD) technique, and tested as anode materials for Li-ion batteries. The electrochemical performance of the as-deposited TG composites were compared to heat-treated TG composites along with pure tin-oxide films. The heat-treated composites exhibited superior specific capacity and energy density than both the as-deposited TG composites and tin oxide samples. At the 70th cycle, the specific capacities of the as-deposited and post heat-treated samples were 534 and 737 mA·h/g, respectively, and the corresponding energy densities of the as-deposited and heat-treated composites were 1240 and 1760 W·h/kg, respectively. This improvement in the electrochemical performance of the TG composite anodes as compared to the pure tin oxide samples is attributed to the synergy between tin oxide and graphene, which increases the electrical conductivity of tin oxide and helps alleviate volumetric changes in tin-oxide during cycling.

## 1. Introduction

To meet the ever increasing energy requirements for modern communication and transportation systems, much research have been invested in improving the performance of lithium-ion batteries (LIBs). One of the most researched areas is the development of novel electrode materials for LIBs, mostly including the usage and development of nanostructures [1,2,3,4]. Due to their high theoretical specific capacity, amorphous tin oxides have been reported as promising candidates for rechargeable LIB anodes [1,5]. It has been proposed that SnO_2_ reacts with lithium and forms Sn and lithium oxide in the first step of the reaction [6,7]. This two-step process is depicted in Equations (1) and (2):

SnO_2_ + 4Li^+^ + 4e^−^ → 2Li_2_O + Sn (1)

Sn + *x*Li^+^ + *x*e^−^ → Li_x_Sn (2)

The Lithium oxide (Li_2_O) formed in the first step of the reaction acts as a buffer matrix during the formation of Li–Sn (Li*_x_*Sn) alloys in the second step of the reaction. Lithium is consumed in the first step, which is in general an irreversible reaction and hence results in the loss of lithium. Besides this loss of lithium, there is a large volumetric expansion in SnO_2_ (>300%) anodes during cycling which results in pulverization of the electrodes and loss of electrical contact. Different methods have been employed to improve the performance of the SnO_2_ anodes such as the use of nanomaterials [8,9], alloys [10,11], and composites [12,13,14]. Tin oxide and graphene composites, for instance, have shown enhanced electrical performance over that of SnO_2_ electrodes [15,16].

The addition of graphene can aid in the improvement of electrical conductivity, thereby resulting in better performance of the electrodes. It can also contribute to the capacity because Li^+^ can adsorb on the surface of the graphene sheet and also along the edges [17]. Graphene has a very high theoretical specific surface area (>2600 m^2^/g). This high specific surface area of graphene increases the accessible area for the electrolyte ions resulting in better charge-transfer kinetics. Graphene can also act as a buffer for volumetric changes during cycling by acting as an inactive matrix when Sn alloys with Li. The literature reporting the use of tin oxide and graphene composites as LIB electrodes has utilized various methods to fabricate these composites including—self-assembly [15], *in situ* chemical synthesis [18], hydrolysis [19], reduction [20], *etc.* Wang *et al.* [16] reported a capacity of 520 mA·h/g for SnO_2_/40% graphene composites after 100 cycles tested in the range of 0.01–3.0 V. For the same composition, Aksay *et al.* [15] reported a capacity of 625 mA·h/g after 100 cycles for anodes tested between 0.02 and 1.5 V. Similarly, for SnO_2_/34% graphene and SnO_2_/36% graphene, the capacities obtained were 634 and 513 mA·h/g after 50 and 10 cycles, respectively and the voltage windows used to test were 0.001–3.0 V and 0.05–3.0 V for SnO_2_/34% graphene and SnO_2_/36% graphene, respectively [18,19]. The discrepancy in the reported electrochemical performances of the composite electrodes can be attributed to several factors such as synthesis method, voltage window, morphology, *etc.*

The fabrication of the electrodes can be simplified by depositing the composite material directly on a current collector. This can be achieved by using electrostatic spray deposition technique (ESD). Samples can be tested without the addition of a binder unlike conventional electrode fabrication techniques. In this work, we have fabricated tin oxide/graphene (TG) composite electrodes using electrostatic spray deposition technique (ESD). Two different sets of composite samples namely as-deposited (195 °C) and post-heat treated (280 °C) were studied along with control samples of pure tin oxide both as-deposited (195 °C) and post-heat treated (280 °C). The samples were characterized and the electrochemical performances were analyzed. It is seen that heat-treated TG composites showed enhanced specific capacity and specific energy density over both the as-deposited TG composites and pure tin-oxide samples. The improvement in electrochemical performance of TG composites as compared to pristine tin oxide samples is ascribed to the synergistic action between tin-oxide and graphene whereas the better performance of the heat-treated samples with respect to as-deposited samples can be attributed to the improvement in crystallinity with heat treatment.

## 2. Results and Discussion

**Figure 1:**
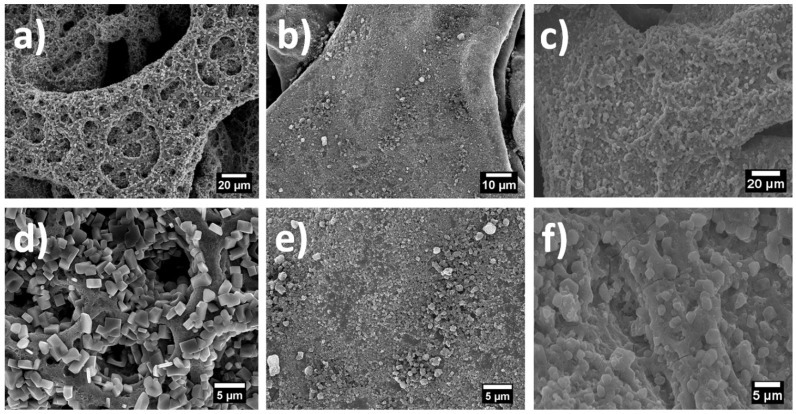
SEM images of (**a**) as-deposited tin oxide; (**b**) as-deposited grapheme; (**c**) as-deposited Tin oxide/graphene (TG) deposited; (**d**) heat-treated tin-oxide; (**e**) heat-treated grapheme; (**f**) heat treated TG composite.

The morphology of the as-deposited (195 °C) and heat-treated (280 °C) films of pure tin oxide, graphene and TG composites are shown in Figure 1. The as-deposited pure tin oxide sample (Figure 1a) shows a porous structure which is typical of an ESD sample deposited from a precursor whereas the as-deposited graphene shows a dense film like morphology, which is expected since no reaction is taking place while deposition. The as-deposited TG composites, on the other hand, do not show a porous structure as expected; the porosity in fact is much less pronounced in TG composites as compared to the as-deposited tin oxide film. This relatively denser morphology can be ascribed to the addition of graphene. From Figure 1d–f, it can be seen that the morphology of the samples is retained after heat treatment and that the heat-treated films show similar structures as their as-deposited counterparts.

**Figure 2:**
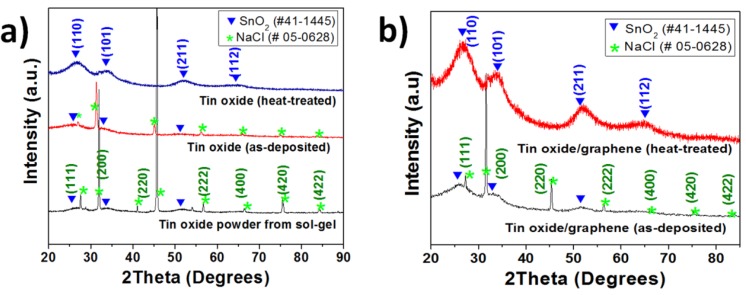
X-ray diffraction patterns of (**a**) tin oxide—the peaks for tin oxide powder obtained from sol-gel, the as-deposited and heat-treated tin oxide are shown; and (**b**) tin oxide/graphene composites—both as-deposited and heat-treated are shown.

Figure 2 shows the X-ray diffraction patterns of the as-deposited and post-heat treated tin oxide samples and those of as-deposited and post-heat treated TG composites. All the samples for XRD were deposited on a glass substrate to avoid the interference from the Ni foam substrate. The peaks at 2θ = 26°, 34°, 52° were assigned to the (110), (101) and (211) planes of SnO_2_ [Joint Committee for Powder Diffraction Standards (JCPDS) card No.: 41-1445] respectively for both pure tin oxide and TG composite samples. The graphene peaks (002) at ~27° are overlapped with tin oxide peaks in the composite samples. Peaks from the residual NaCl in the tin oxide powder used in the precursor solution can be observed in the patterns (JCPDS card No.: 05-0628) for both as-deposited tin oxide and the TG composite samples. However, these NaCl peaks are not seen for the heat-treated films, indicating that the heat-treated samples had higher purity than the as-deposited samples.

**Figure 3:**
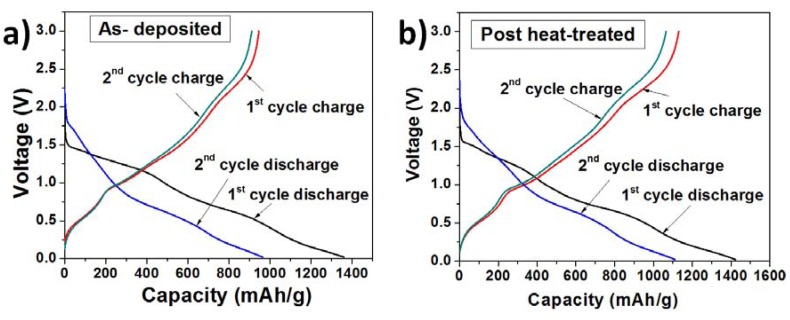
Charge-discharge profiles of (**a**) TG composites at 195 °C (as deposited); and (**b**) TG composites at 280 °C (post-heat treated).

The charge-discharge profiles of the as-deposited and heat-treated TG composites (Figure 3) were obtained using cyclic charge-discharge at a constant current density of 0.2 C between 0.01 and 3.0 V. The profiles show three voltage plateaus corresponding to the decomposition reaction (~1.25 V), lithium alloy formation with tin (~0.5 V) and graphene (~0.1 V) during discharge. During charging, the lithium de-alloys from tin around ~0.75 V and lithium de-intercalates from graphene layers around 0.3 V. There is no imperceptible difference in the voltage plateaus for the as-deposited composites and post-treat heated samples. The first cycle discharge capacity of the as-deposited TG composite was 1360 mA·h/g whereas the charge capacity was 940 mA·h/g, resulting in a Columbic efficiency of 69%. For the second cycle, the charge capacity was 915 mA·h/g, however the discharge capacity reduced to 964 mA·h/g, resulting in an increased columbic efficiency of 95%. The capacity loss between the first and second discharge cycles was ~396 mA·h/g. For the heat-treated TG composite, the first and second cycle discharge capacities were 1423 and 1110 mA·h/g, making a discharge capacity loss of ~313 mA·h/g. The charge capacities were 1124 mA·h/g and 1065 mA·h/g for the first and second cycles and the corresponding columbic efficiencies were 79% and 96%. The increase in the columbic efficiency suggests that the number of Li^+^ ions that were trapped or the ones undergoing side reactions reduced considerably after the first cycle.

**Figure 4:**
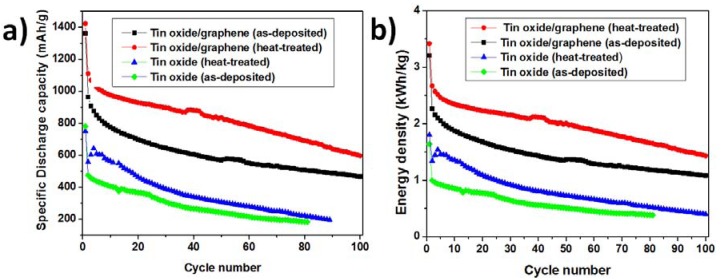
Comparison of (**a**) cycle performance and (**b**) energy density of tin oxide/graphene (TG) composites and tin oxide samples for both as-deposited and heat treated samples.

The capacity and cycle performances of the electrodes were evaluated by galvanostatic discharge-charge measurements at a discharge rate of 0.2 C (Figure 4a). Both the composite electrodes showed a gradual decrease in the capacity. For the as-deposited and heat treated tin oxide anodes, the initial capacities were around 780 and 750 mA·h/g, respectively. The irreversible capacity loss was ~200 mA·h/g for the heat-treated tin oxide anode and 310 mA·h/g for the as-deposited tin oxide anode. At the 10th cycle, the as-deposited TG composite showed a capacity of 774 mA·h/g, whereas the heat treated TG composite showed a capacity of 975 mA·h/g. All of the electrodes lose their capacities with increasing number of cycles. After 70 cycles, the as-deposited and heat-treated TG composites retained 55% and 66% of the second cycle capacity respectively, whereas the as-deposited and heat-treated tin-oxide electrodes only retained 33% and 42%, respectively. The effect of the addition of graphene to tin oxide can be clearly seen from Figure 3. The capacity increased more than two times upon the addition of graphene, which improves the conductivity of tin oxide and acts as a buffer matrix during cycling.

The energy density of the anodes (Figure 3b) was calculated using the following formula:

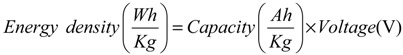
(3)
where Voltage (V) is the voltage window.

At the 70th cycle, the energy density of the as-deposited and heat treated TG composites was 1240 and 1760 W·h/kg. These values are much higher than those of the commercially used graphite anodes, which usually have energy densities in the range of 37.2–74.4 W·h/kg [21].

**Figure 5:**
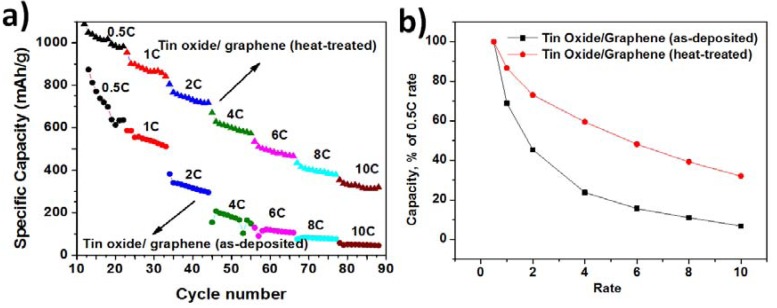
(**a**) Rate capability of TG composites—as-deposited and heat-treated; and (**b**) Normalized capacity *vs.* the rate of discharge of TG composites—as-deposited and heat treated.

Figure 5 shows the rate capability of the composite anodes and normalized capacities with respect to the capacity obtained at 0.5 C. The samples were tested at 0.5 C, 1 C, 2 C, 4 C, 6 C, 8 C, and 10 C rates. The rate capability of heat treated TG composite was better than the as-deposited TG composite. The degradation rates of specific discharge capacities with increasing current density were lower for composites at 280 °C. However, both the composites lose their capacity with increasing rate of discharge. At a discharge rate of 10 C, the capacity obtained was only 30% of the capacity obtained at 0.5 C rate for the heat-treated TG composites, whereas it was only 10% for the as-deposited TG composite.

From the above figures and discussions, it can be concluded that (a) The TG composites although had a porous structure but the porosity was not as pronounced as that in tin oxide films; (b) XRD patterns for TG composites showed SnO_2_ peaks along with graphene peaks. In addition, peaks corresponding to NaCl were observed, indicating the remnant impurity during the sol-gel process. These peaks were not observed for the heat-treated samples, indicating higher levels of purity in the heat-treated samples; (c) The Columbic efficiencies increased in the second cycles for both TG composites at 195 °C and 280 °C indicating a decrease in the number of trapped Li^+^ and/or the Li^+^ ions undergoing side reactions; (d) From Figure 4, it is seen that the TG composites at 280 °C exhibit better cycle performance and energy density than both the TG composites at 195 °C and the tin oxide samples; (e) TG composites at 280 °C showed superior rate capability and as well as normalized capacity than the TG composites deposited at 195 °C.

## 3. Experimental Section

0.108 M NaOH was added slowly to 0.054 M SnCl_4_. Both the chemicals were purchased from Sigma Aldrich. The resulting sol solution was heated until all the solvent evaporated, leaving behind a white powder. This powder was then used in the preparation of the precursor solution. The precursor solution for tin oxide/graphene composites (TG composites) was prepared by mixing tin oxide and 30 wt.% graphene (Cheaptubes Inc., Brattleboro, VT, USA) with ethylene glycol (Sigma Aldrich, St. Louis, MO, USA) and ethanol (Sigma Aldrich, St. Louis, MO, USA). This solution was then deposited on a nickel foam substrate (Hunan Corun New Energy Co. Ltd, Changsha, China) by using electrostatic spray deposition (ESD) technique at a temperature of 195 °C. Some of the samples deposited were post-heat treated at a temperature of 280 °C in an N_2_ atmosphere. The morphologies of the as-deposited films were investigated using Scanning electron microscopy (JEOL 6335 FE-SEM, Peabody, MA, USA). X-ray diffraction (XRD) studies were carried out using the D5000 diffracktometer. Electrochemical test cells were assembled in an argon atmosphere using TG thin film samples as anode and lithium as counter and reference electrode in an electrolyte of 1.1M FC-130 dissolved in ethylene carbonate, dimethyl carbonate and diethyl carbonate (EC:DMC:DEC, 1:1:1 *w*/*w*/*w*). Electrochemical tests were carried out between 3.00 and 0.02 V at a rate of 0.2 C.

## 4. Conclusions

Tin oxide and graphene composites were fabricated using Electrostatic Spray Deposition (ESD) and their electrochemical performance was studied. The composites showed better performance than the pure tin oxide anodes synthesized under the same experimental conditions. Both the specific capacity and energy density of the heat-treated TG composite were higher than that of both the as-deposited TG composite and pure tin-oxide samples. The specific capacities of the as-deposited and post heat-treated composite samples were 534 and 737 mA·h/g, respectively at the 70th cycle and the corresponding energy densities of the as-deposited and heat-treated composites were 1240 and 1760 W·h/kg, respectively. This enhancement in electrochemical behavior of tin oxide and graphene composites relative to pure tin oxide films is attributed to the synergistic effect from the addition of graphene. The better performance of the heat-treated samples with respect to their as-deposited counterparts can be ascribed to the improvement in crystallinity of tin oxide, after the heat-treatment. Moreover, the heat-treated samples show no remnant NaCl peaks in XRD patterns, which suggests higher levels of purity in heat-treated samples and can be another reason for better performance.
